# Molecular characterization and clonal evolution in Richter transformation: Insights from a case of plasmablastic lymphoma (RT‐PBL) arising from chronic lymphocytic leukaemia (CLL) and review of the literature

**DOI:** 10.1002/jha2.771

**Published:** 2023-08-18

**Authors:** Megan C. Ramsey, Peter J. B. Sabatini, Adam C. Smith, Ali Sakhdari

**Affiliations:** ^1^ Laboratory Medicine Program Toronto General Hospital University Health Network Toronto Ontario Canada; ^2^ Department of Laboratory Medicine and Pathobiology Temerty Faculty of Medicine University of Toronto Toronto Ontario Canada

1

Richter transformation (RT) represents a high‐grade transformation observed in chronic lymphocytic leukaemia (CLL)/small lymphocytic lymphoma, leading to increased aggressiveness and unfavourable outcomes. Among the variants of RT, diffuse large B‐cell lymphoma (RT–DLBCL) is the most commonly encountered, whereas transformation into plasmablastic lymphoma (RT–PBL) is an exceptionally rare event [1, 2]. PBL is characterized by the presence of large atypical B cells displaying plasmablastic or immunoblastic morphology and exhibiting a terminal B‐cell differentiation phenotype. Typically, PBL arises de novo in patients with immune deficiency or dysregulation [2, 3]. In this context, we report a compelling case of RT in a patient with unmutated CLL who experienced transformation to PBL 14 months following the initial diagnosis.

A 71‐year‐old man presented with new‐onset lymphocytosis (17.9–22.7 × 10^9/L), without B symptoms or lymphadenopathy. Flow cytometry confirmed a CLL immunophenotype. After 2 months, the patient experienced fatigue, decreased appetite and weight loss, with a significant increase in lymphocyte count (76 × 10^9/L). Radiological investigation revealed mesenteric, retroperitoneal and pelvic lymphadenopathy. Bone marrow biopsy revealed 90% involvement by CLL. There was no histological evidence of transformation on bone marrow or peripheral blood (Figure 1). The patient was started on Ibrutinib treatment. He remained stable for 12 months, and his lymphocyte count normalized to 2.7 × 10^9/L. Subsequently, he presented with a 2‐week history of left leg swelling and immobility. A CT scan revealed extensive lymphadenopathy, including a 15.6 cm conglomerate mass involving abdominal organs. A lymph node biopsy showed effacement by large cells with abundant pale cytoplasm, vesicular nuclei and prominent nucleoli (Figure 1). Mitoses and apoptosis were abundant. Immunohistochemical analysis demonstrated positive staining for plasmacytic markers CD138 and MUM‐1 and a proliferation index close to 100% (Figure 2). HHV8 and Epstein–Barr virus (EBV) were negative. A summary of the immunohistochemical features for both diagnoses is provided in Table S1.

**FIGURE 1:**
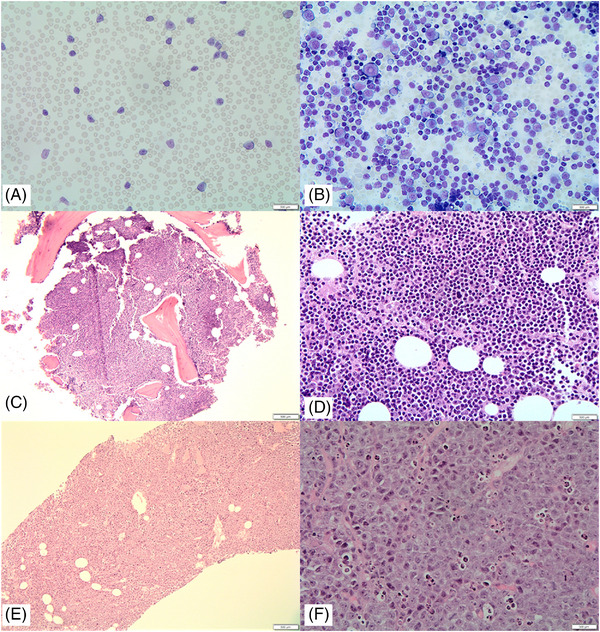
(A) Peripheral blood film (40×) with circulating chronic lymphocytic leukaemia (CLL) cells; (B) bone marrow aspirate (40×), with a predominant infiltrate of small CLL cells and decreased background haematopoiesis; (C and D) bone marrow trephine (10× and 40×, *ha*ematoxylin and *e*osin) with a diffuse infiltrate of small lymphoid cells representing 90% of marrow cellularity; (E and F) lymph node biopsy (10× and 40×, *ha*ematoxylin and *e*osin) with diffuse effacement by large lymphoid cells with pale cytoplasm, vesicular nuclei with prominent nucleoli and abundant apoptosis.

**FIGURE 2:**
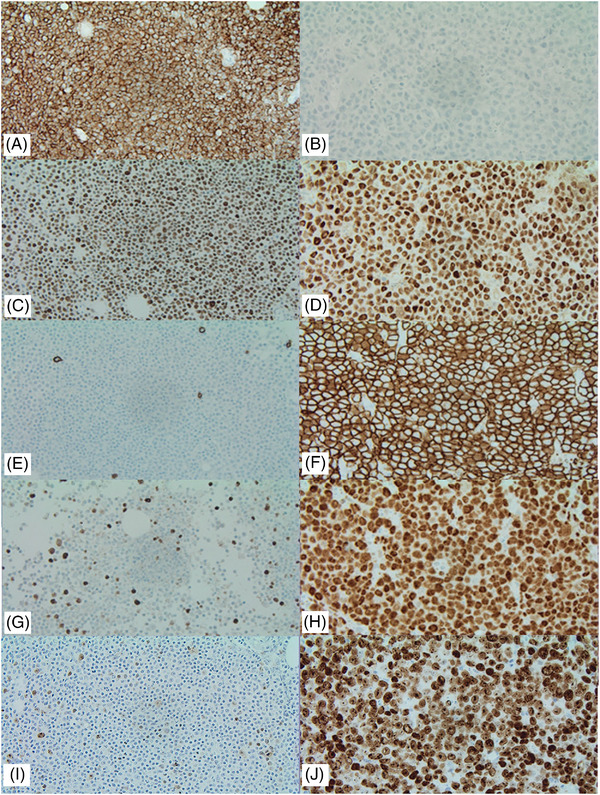
(A and B) CD20, chronic lymphocytic leukaemia (CLL) and plasmablastic lymphoma (PBL), respectively; (C and D) LEF1, CLL and PBL, respectively; (E and F) CD138, CLL and PBL, respectively; (G and H) C‐Myc, CLL and PBL, respectively; (I and J) MKi67, CLL and PBL, respectively (all 40× magnifications).

Due to multifactorial medical complications, the patient required ICU admission shortly after. Two cycles of CHOP chemotherapy were administered at reduced dosages but soon switched to palliative treatment. The patient passed away 4 months after RT and 19 months after the initial workup for lymphocytosis.

*IGH*V mutation status testing was conducted on the bone marrow biopsy with CLL and the lymph node biopsy with PBL. The CLL and PBL populations were found to have identical clonal IGH gene rearrangements (IGHV3‐30‐301, IGHJ6‐02, IGHD3‐3*01) that were unmutated (100% homology to IGHV IGMT reference set) [4]. FISH testing with a CLL prognostic panel [5] was performed on the bone marrow with CLL which showed the signal pattern for the centromere of chromosome 12 (CEP12)(D12Z1) probe consistent with trisomy 12 in 68% of nuclei. CEP12 FISH analysis on the PBL was also positive. FISH testing with *MYC, BCL2* and *BCL6* break‐apart probes was conducted on PBL only. The *MYC* and *BCL2* break‐apart probes were positive, whereas a subsequent FISH with the dual fusion *IGH/BCL2* probe was negative indicating a non‐IGH partner.

Next‐generation sequencing (NGS) was performed on both samples using the Illumina TruSight Oncology 500 (TSO500) targeted hybrid‐capture‐based NGS assay covering a comprehensive list of 500 genes (Supporting Information Data 1) [6]. Sequencing analysis of CLL revealed two sequence variants. The *NOTCH1* gene variant (NM_017617.5): c.7375C > T (p.Gln2459Ter) was detected at a frequency of 44%, and the *SPEN* gene variant (NM_015001.3): c.5920dupA (p.Thr1974fsTer6) was identified at a frequency of 42%. In PBL, the same sequence variants of *NOTCH1* and *SPEN* were present at similar frequencies of 47% and 43%, respectively (Table S2). The analysis revealed the presence of copy number variants, specifically amplifications, in the *BRAF*, *CDK6*, *EGFR* and *MET* genes within PBL. Additionally, sequence variants were identified in *ARID1B*, *BCL2* and *ERBB2* (Table S3).

A review of the literature on PBL arising in the context of CLL yielded 15 cases [2, 7–16], the findings of which are summarized in Table S4. The male‐to‐female ratio was 4:1 and age at primary CLL diagnosis ranged from 52 to 77 years. None of the patients had a known history of immunodeficiency. Three cases had both CLL and PBL diagnosed simultaneously [2, 10, 11], whereas for the remaining 12 cases, the time between CLL diagnosis and PBL diagnosis varied from 14 to 132 months. Four patients had received prior treatment with Ibrutinib for a duration of 18–96 months before developing plasmablastic lymphoma. In 10 out of 12 cases, a clonal relationship between CLL and PBL was established. The two cases that were clonally unrelated according to IGH sequencing were attributed to PBL development as a secondary lymphoma due to immunosuppression resulting from previous CLL treatments (fludarabine and cladribine, respectively) [15, 16]. The interval between PBL diagnosis and death was generally short, with 12 patients succumbing within 6 months. Of the 14 tested cases, 11 were negative for EBV, and 3 were positive.

FISH analysis was conducted on nine cases of CLL [2, 7–9], with the most common alterations being 17p13.1 abnormalities (4/9), followed by 13q14.3 deletions (3/9). Trisomy 12 was detected in two cases, and 11q22 deletion was found in one. One case exhibited trisomy 12 and a 17p13.1 deletion, along with a *TP53* mutation.^14^ In two cases, FISH analysis was performed for *BCL2, BCL6, CCND1*, *MYC* and *TP53* abnormalities in both the CLL and PBL. One CLL case had a t(12;14). rearrangement with *MYC* rearrangement at transformation, whereas the second case had an *MYC* rearrangement, which persisted in the subsequent PBL [7]. Four of seven cases that underwent *MYC* rearrangement studies on PBL were positive. *BCL2* and *BCL6* FISH studies were performed on five cases, revealing gains in *BCL2* and *BCL6* in two and three cases, respectively. The results of NGS analysis performed on five cases are summarized in Table S5.

Although reviewing the literature revealed that *TP53* abnormalities were the most common genetic change in RT–PBL with no *NOTCH1* mutation previously reported, this case showed *NOTCH1* and *SPEN* mutations but not *TP53*. *MYC* rearrangements are found in both de novo PBL and RT–PBL indicating their significant role in disease progression [14]. In this review, five patients developed RT–PBL after ibrutinib, raising the possibility that CLL transforms to RT–PBL as a mechanism of resistance to BCR inhibition or that minor RP–PBL subclones might have been present at treatment initiation potentially selected with a BCR inhibition. A notable distinguishing factor between RT and de novo PBL is the absence of EBV in most cases of RT, whereas de novo PBL is typically EBV‐positive [17].

In conclusion, our case adds to the understanding of the genetic and molecular characteristics of PBL as RT. Not many studies have shown a distinct genetic or molecular abnormality that differentiates RT–PBL from RT–DLBCL or de novo PBL [18], although this needs further large cohort analysis [19, 20]. However, our case demonstrated the concurrence of two mutually exclusive genetic pathways observed in CLL to DLBCL transformations: the *TP53* mutation with *MYC* activation pathway and trisomy 12 with *NOTCH1* mutated pathway [21]. Further investigations, including NGS analysis of PBL as RT cases, are warranted to identify unique genetic factors contributing to the development of PBL as RT over DLBCL.

## AUTHOR CONTRIBUTIONS

*Study design; acquisition; assembly; analysis and interpretation of data; drafting of the manuscript; critical revision of the manuscript for important intellectual content*: Ali Sakhdari. *Study design; acquisition; interpretation of data; drafting of the manuscript; critical revision of the manuscript*: Megan C. Ramsey. *Acquisition; analysis; and interpretation of molecular data; drafting of the manuscript; critical revision of the manuscript*: Peter J. B. Sabatini and Adam C. Smith.

## CONFLICT OF INTEREST STATEMENT

The authors declare that they have no conflicts of interest.

## FUNDING INFORMATION

The authors received no specific funding for this work.

## ETHICS STATEMENT

This study followed the University Health Network and patients’ ethics. The study was approved by the “Research Ethics Board [REB]” of the University Health Network and conducted in compliance with the Declaration of Helsinki.

## CLINICAL TRIAL REGISTRATION

The authors have confirmed clinical trial registration is not needed for this submission.

## PATIENT CONSENT STATEMENT

The authors have confirmed patient consent statement is not needed for this submission.

## Supporting information

Supporting Information

## Data Availability

The data that support the findings of this study are available on request from the corresponding author. The data are not publicly available due to privacy or ethical restrictions.
