# Supplementary material for: Molecular characterization and clonal evolution in Richter transformation: Insights from a case of plasmablastic lymphoma (RT‐PBL) arising from chronic lymphocytic leukaemia (CLL) and review of the literature
Source: EJHaem. 2023 Aug 18;4(4):1203–7. doi: 10.1002/jha2.771 (PMC10660098; doi:10.1002/jha2.771)
Supplement: Supplementary file 1 — Supporting Information [file JHA2-4-1203-s001.docx]

Supplementary Tables and data

Supplementary Table 1. Immunohistochemical features of chronic lymphocytic leukemia (CLL) in the bone marrow versus the plasmablastic lymphoma (PBL) in the lymph node.

| Stain | CLL | PBL |
| --- | --- | --- |
| CD5 | Positive | Negative |
| CD20 | Positive | Negative |
| CD23 | Positive | Negative |
| CD30 | Negative | Positive; subset 10-20% |
| CD138 | Negative | Positive |
| BCL-2 | Positive; diffuse strong | Positive; diffuse, strong |
| CCND1 | Negative | Negative |
| C-MYC | 10%, strong | Positive; diffuse, strong |
| MUM-1 | 10%, weak | Positive; diffuse, strong |
| LEF-1 | Positive | Positive |
| Pax-5 | Positive, Moderate, diffuse | Negative |
| IG Kappa | Inconclusive ^a^ | Positive |
| IG Lambda | Inconclusive | Negative |
| Ki-67 | ~10% | ~100% |
| Other |  | ALK1 negative  BCL6 negative  CD56 10% weak positive  CD117 negative  p53 negative |

^a^ The interpretation of IG kappa and IG lambda in the CLL was inconclusive due to background staining. The stains highlighted a small background population of polytypic plasma cells. By flow cytometry, the CLL cells had dim surface IG lambda.

Supplementary Table 2. Shared sequence variants detected in the chronic lymphocytic leukemia (CLL) and the plasmablastic lymphoma (PBL) of current case.

| CLL | PBL |
| --- | --- |
| NOTCH1 (NM_017617.5): c.7375C>T (p.Gln2459Ter) 44%  SPEN (NM_015001.3): c.5920dupA (p.Thr1974fsTer6) 42% | NOTCH1 (NM_017617.5): c.7375C>T (p.Gln2459Ter) 47%  SPEN (NM_015001.3): c.5920dupA (p.Thr1974fsTer6) 43% |

Supplementary Table 3. List of unique variants detected in lymphocytic leukemia (CLL) and plasmablastic lymphoma (PBL) in the current case.

| CLL | PBL |
| --- | --- |
| BCL2 (NM_000633.3): c.66G>A (p.Lys22Lys) 7.41% | BRAF (NM_004333.6): amplification (copy number = 5)  EGFR (NM_005228.5): amplification (copy number = 4)  MET (NM_001127500.3): amplification (copy number = 5)  CDK6 (NM_001259.8): amplification (copy number = 4)  ARID1B (NM_001374820.1): c.1337C>T (p.Ala446Val) 67%  BCL2 (NM_000633.3): c.175C>G (p.Pro59Ala) 46%  BCL2 (NM_000633.3): c.392C>A (p.Ala131Asp) 49%  ERBB2 (NM_004448.4): c.2382G>A (p.Val794Val) 45% |

Supplementary Table 4. Summary of the clinical features in the cases of Richter transformation (RT) to plasmablastic lymphoma in the literature in comparison to the case presented here.

| Case Reference | Gend | Age at diag. | IDD | Ibrutinib/  duration (m) | time to  RT (m) | Site of RT | Clonally related  (CLL/ PBL) | RT diagnosis | EBV | Clinical outcome |
| --- | --- | --- | --- | --- | --- | --- | --- | --- | --- | --- |
| Current case | M | 71 | No | Y,12 | 14 | Lymph node | Y | PBL | Neg (EBER-ISH) | D,  19 m |
| Gángó A et al. ^7^ | M | 75 | No | Y,18 | 52 | Gingiva | NS | PBL | NS | D,  53 m |
| Marvyin K et al. ^8^ | M | 53 | No | Y,24 | 93 | Lymph node | Y | PBL | Neg (EBER-ISH) | D,  94 m |
| Gasljevic G et al. ^9^ | F | 74 | No | N | 132 | Lymph node | Y | Composite  CHL and PBL | Neg (EBER-ISH) | D,  132 m |
| Hatzimichael E et al. ^10^ | M | 67 | No | N | 0 | Lymph node,  bone & GI | NS | CLL and PBL | Pos (EBER-ISH) | D,  2.5 m |
| Ronchi A et al. ^11^ | M | 61 | No | N | 0 | Lymph node | Y | CLL and PBL | Neg (EBER-ISH) | D,  NOS |
| Chan KL et al ^12^ | M | 63 | No | Y, 96 | 102 | GI | Y | PBL | Neg (EBER-ISH) | D,  102 m |
| Chan KL et al ^12^ | M | 67 | No | Y, 84 | 89 | Lymph node | Y | PBL | Neg (EBER-ISH) | D,  92 m |
| Evans AG et al. ^13^ | M | 62 | No | N | 48 | Gingiva | Y | PBL | Neg (EBER-ISH) | D,  49 m |
| Pan Z et al. ^14^ | M | 58 | No | N | 96 | Lymph node, spleen | Y | PBL | Neg (EBER-ISH) | D,  99 m |
| Pan Z et al. ^14^ | M | 77 | No | N | 60 | Bone marrow | Y | PBL | Neg (EBER-ISH) | Alive, 108 m |
| Foo WC et al ^15^ | M | 69 | No | N | 37 | Nasopharynx | N | Composite  CHL and PBL | Pos (EBER-ISH) | D,  46 m |
| Robak T et al. ^16^ | F | 57 | No | N | 48 | Mandible | N | PBL | Neg (LMP1-PCR) | D,  54 m |
| Martinez D et al ^2^ | M | 70 | No | N | 0 | Lymph node | Y | CLL in Bone marrow, PBL Lymph node | Neg (EBER-ISH) | D,  4 m |
| Martinez D et al ^2^ | M | 52 | No | N | 85 | Sub-cutaneous | Y | PBL | Pos (EBER-ISH)/ Neg (LMP1-PCR) | D,  109 m |
| Martinez D et al ^2^ | F | 57 | No | N | 47 | Mandible | NS | PBL | Neg (EBER-ISH) | D,  53 m |

Abbreviation: CLL, Chronic Lymphocytic Leukemia; D, Dead; F, female; GI, gastrointestinal; IDD, Immunodeficiency/dysregulation; m, month; M, Male; N, No; Neg, Negative; NS, not specified; PBL, Plasmablastic lymphoma; Pos, Positive; RT, Richter Transformation; Y, Yes.

Supplementary Table 5. Summary of sequencing results in chronic lymphocytic leukemia and/or plasmablastic lymphoma.

| Case reference | Variant detected (CLL) | VAF | Variant detected (PBL) | VAF |
| --- | --- | --- | --- | --- |
| Gángó A et al. ^7^ | *TP53*: p.E258Pfs*85 | 8.5% | Negative |  |
| Marvyin K et al. ^8^ | *TP53*: c.329G>C;p.Arg110Pro | NS | NP |  |
| Chan KL et al. ^12^ | *TP53*: c.731G>A;p.Gly244Asp | NS | *TP53*: c.731G>A; p.Gly244Asp | NS |
|  |  |  | *NRAS*: c.35G>T; p.Gly12Val | NS |
| Chan KL et al. ^12^ | *TP53*: c.817C>A; p.Arg273Ser | NS | NP |  |
|  | *NRAS:* c.38G>T; p.Gly13Val | NS |  |  |
| Evans AG et al. ^13^ | *TP53*: p.Gly245Ser; p.Val197Glu | NS | *TP53*: p.Gly245Ser | NS |

Abbreviations: NP, not performed; NS, not specified; VAF, variant allele fraction

Supplementary Data

Supplementary data 1. List of all the genes tested using the using the Illumina TruSight™ Oncology 500 (TSO500) targeted hybrid-capture based next generation sequencing assay.

Sequence Variants

*ABL1, ABL2, ACVR1, ACVR1B, AKT1, AKT2, AKT3, ALK, ALOX12B, ANKRD11, ANKRD26, APC, AR, ARAF, ARFRP1, ARID1A, ARID1B, ARID2, ARID5B, ASXL1, ASXL2, ATM, ATR, ATRX, AURKA, AURKB, AXIN1, AXIN2, AXL, B2M, BAP1, BARD1, BBC3, BCL10, BCL2, BCL2L1, BCL2L11, BCL2L2, BCL6, BCOR, BCORL1, BCR, BIRC3, BLM, BMPR1A, BRAF, BRCA1, BRCA2, BRD4, BRIP1, BTG1, BTK, C11orf30, CALR, CARD11, CASP8, CBFB, CBL, CCND1, CCND2, CCND3, CCNE1, CD274, CD276, CD74, CD79A, CD79B, CDC73, CDH1, CDK12, CDK4, CDK6, CDK8, CDKN1A, CDKN1B, CDKN2A, CDKN2B, CDKN2C, CEBPA, CENPA, CHD2, CHD4, CHEK1, CHEK2, CIC, CREBBP, CRKL, CRLF2, CSF1R, CSF3R, CSNK1A1, CTCF, CTLA4, CTNNA1, CTNNB1, CUL3, CUX1, CXCR4, CYLD, DAXX, DCUN1D1, DDR2, DDX41, DHX15, DICER1, DIS3, DNAJB1, DNMT1, DNMT3A, DNMT3B, DOT1L, E2F3, EED, EGFL7, EGFR, EIF1AX, EIF4A2, EIF4E, EML4, EP300, EPCAM, EPHA3, EPHA5, EPHA7, EPHB1, ERBB2, ERBB3, ERBB4, ERCC1, ERCC2, ERCC3, ERCC4, ERCC5, ERG, ERRFI1, ESR1, ETS1, ETV1, ETV4, ETV5, ETV6, EWSR1, EZH2, FAM123B, FAM175A, FAM46C, FANCA, FANCC, FANCD2, FANCE, FANCF, FANCG, FANCI, FANCL, FAS, FAT1, FBXW7, FGF1, FGF10, FGF14, FGF19, FGF2, FGF23, FGF3, FGF4, FGF5, FGF6, FGF7, FGF8, FGF9, FGFR1, FGFR2, FGFR3, FGFR4, FH, FLCN, FLI1, FLT1, FLT3, FLT4, FOXA1, FOXL2, FOXO1, FOXP1, FRS2, FUBP1, FYN, GABRA6, GATA1, GATA2, GATA3, GATA4, GATA6, GEN1, GID4, GLI1, GNA11, GNA13, GNAQ, GNAS, GPR124, GPS2, GREM1, GRIN2A, GRM3, GSK3B, H3F3A, H3F3B, H3F3C, HGF, HIST1H1C, HIST1H2BD, HIST1H3A, HIST1H3B, HIST1H3C, HIST1H3D, HIST1H3E, HIST1H3F, HIST1H3G, HIST1H3H, HIST1H3I, HIST1H3J, HIST2H3A, HIST2H3C, HIST2H3D, HIST3H3, HLA-A, HLA-B, HLA-C, HNF1A, HNRNPK, HOXB13, HRAS, HSD3B1, HSP90AA1, ICOSLG, ID3, IDH1, IDH2, IFNGR1, IGF1, IGF1R, IGF2, IKBKE, IKZF1, IL10, IL7R, INHA, INHBA, INPP4A, INPP4B, INSR, IRF2, IRF4, IRS1, IRS2, JAK1, JAK2, JAK3, JUN, KAT6A, KDM5A, KDM5C, KDM6A, KDR, KEAP1, KEL, KIF5B, KIT, KLF4, KLHL6, KMT2B, KMT2C, KMT2D, KRAS, LAMP1, LATS1, LATS2, LMO1, LRP1B, LYN, LZTR1, MAGI2, MALT1, MAP2K1, MAP2K2, MAP2K4, MAP3K1, MAP3K13, MAP3K14, MAP3K4, MAPK1, MAPK3, MAX, MCL1, MDC1, MDM2, MDM4, MED12, MEF2B, MEN1, MET, MGA, MITF, MLH1, MLL, MLLT3, MPL, MRE11A, MSH2, MSH3, MSH6, MST1, MST1R, MTOR, MUTYH, MYB, MYC, MYCL1, MYCN, MYD88, MYOD1, NAB2, NBN, NCOA3, NCOR1, NEGR1, NF1, NF2, NFE2L2, NFKBIA, NKX2-1, NKX3-1, NOTCH1, NOTCH2, NOTCH3, NOTCH4, NPM1, NRAS, NRG1, NSD1, NTRK1, NTRK2, NTRK3, NUP93, NUTM1, PAK1, PAK3, PAK7, PALB2, PARK2, PARP1, PAX3, PAX5, PAX7, PAX8, PBRM1, PDCD1, PDCD1LG2, PDGFRA, PDGFRB, PDK1, PDPK1, PGR, PHF6, PHOX2B, PIK3C2B, PIK3C2G, PIK3C3, PIK3CA, PIK3CB, PIK3CD, PIK3CG, PIK3R1, PIK3R2, PIK3R3, PIM1, PLCG2, PLK2, PMAIP1, PMS1, PMS2, PNRC1, POLD1, POLE, PPARG, PPM1D, PPP2R1A, PPP2R2A, PPP6C, PRDM1, PREX2, PRKAR1A, PRKCI, PRKDC, PRSS8, PTCH1, PTEN, PTPN11, PTPRD, PTPRS, PTPRT, QKI, RAB35, RAC1, RAD21, RAD50, RAD51, RAD51B, RAD51C, RAD51D, RAD52, RAD54L, RAF1, RANBP2, RARA, RASA1, RB1, RBM10, RECQL4, REL, RET, RFWD2, RHEB, RHOA, RICTOR, RIT1, RNF43, ROS1, RPS6KA4, RPS6KB1, RPS6KB2, RPTOR, RUNX1, RUNX1T1, RYBP, SDHA, SDHAF2, SDHB, SDHC, SDHD, SETBP1, SETD2, SF3B1, SH2B3, SH2D1A, SHQ1, SLIT2, SLX4, SMAD2, SMAD3, SMAD4, SMARCA4, SMARCB1, SMARCD1, SMC1A, SMC3, SMO, SNCAIP, SOCS1, SOX10, SOX17, SOX2, SOX9, SPEN, SPOP, SPTA1, SRC, SRSF2, STAG1, STAG2, STAT3, STAT4, STAT5A, STAT5B, STK11, STK40, SUFU, SUZ12, SYK, TAF1, TBX3, TCEB1, TCF3, TCF7L2, TERC, TERT, TET1, TET2, TFE3, TFRC, TGFBR1, TGFBR2, TMEM127, TMPRSS2, TNFAIP3, TNFRSF14, TOP1, TOP2A, TP53, TP63, TRAF2, TRAF7, TSC1, TSC2, TSHR, U2AF1, VEGFA, VHL, VTCN1, WISP3, WT1, XIAP, XPO1, XRCC2, YAP1, YES1, ZBTB2, ZBTB7A, ZFHX3, ZNF217, ZNF703, ZRSR2*

Focal Amplifications

*AKT2, ALK, AR, ATM, BRAF, BRCA1, BRCA2, CCND1, CCND3, CCNE1, CDK4, CDK6, CHEK1, CHEK2, EGFR, ERBB2, ERBB3, ERCC1, ERCC2, ESR1, FGF1, FGF10, FGF14, FGF19, FGF2, FGF23, FGF3, FGF4, FGF5, FGF6, FGF7, FGF8, FGF9, FGFR1, FGFR2, FGFR3, FGFR4, JAK2, KIT, KRAS, LAMP1, MDM2, MDM4, MET, MYC, MYCL1, MYCN, NRAS, NRG1, PDGFRA, PDGFRB, PIK3CA, PIK3CB, PTEN, RAF1, RET, RICTOR, RPS6KB1, TFRC*

Fusions (from RNA)

*ALK, AR, BRAF, BRCA1, BRCA2, CDK4, EGFR, ERBB2, ESR1, FGFR1, FGFR2, FGFR3, FGFR4, JAK2, KIT, MET, MYC, NRG1, PDGFRA, PDGFRB, PIK3CA, RAF1, RET, RPS6KB1, ABL1, AKT3, AXL, BCL2, CSF1R, EML4, ERG, ETS1, ETV1, ETV4, ETV5, EWSR1, FLI1, FLT1, FLT3, KDR, KIF5B, MLL, MLLT3, MSH2, NOTCH1, NOTCH2, NOTCH3, NTRK1, NTRK2, NTRK3, PAX3, PAX7, PPARG, ROS1, TMPRSS2*
